# Beyond borders: investigating the impact of the 2023 conflict in Gaza on generalized anxiety disorders and associated somatic symptoms and wellbeing among the Kuwait population: a global call for public intervention programs

**DOI:** 10.3389/fpubh.2025.1407906

**Published:** 2025-02-12

**Authors:** Musaed Z. Alnaser, Hamad Alhamad, Naser Alotaibi, Nadia Alhamdan, Fahad Manee

**Affiliations:** ^1^Occupational Therapy Department, Faculty of Allied Health Sciences, Kuwait University, Kuwait City, Kuwait; ^2^Psychology Department, College of Social Sciences, Kuwait University, Kuwait City, Kuwait

**Keywords:** mental health, armed conflict, war, news, fear, social media

## Abstract

**Purpose:**

Armed conflicts can lead to devastating psychological health issues within and around conflict zones. Generalized anxiety disorder, associated somatic symptoms, and wellbeing were examined among the Kuwait population during the Gaza Conflict in 2023, in addition to exploring the provoking factors.

**Methods:**

A descriptive, correlational cross-sectional design was used in this study. An online survey was conducted to collect information from people living in Kuwait during the Gaza Conflict. Data were collected between November 20 and November 27, 2023. The generalized anxiety disorder scale (GAD-7), a modified patient health questionnaire (mPHQ-15), and the World Health Organization – Five (WHO-5) were used to examine self-reported anxiety, somatic symptoms, and wellbeing among the study participants. Spearman’s correlation and chi-squared tests were used to determine the relationships between anxiety, somatic symptoms, and wellbeing, and to capture the instigating factors.

**Results:**

This study included 1979 participants living in Kuwait during the Gaza conflict period. GAD-7 and mPHQ-5 final scores were moderate (*μ* = 10.20 ± 4.48; *μ* = 11.12 ± 6.39), and WHO-5 final score was fair (*μ* = 53.35 ± 21.82). Spearman’s correlation showed a large positive correlation between the GAD-7 and mPHQ-15, *r*(1977) = 0.52, *p* < 0.0001, a medium negative correlation between the GAD-7 and WHO-5, *r*(1977) = −0.36, *p* < 0.0001, and a medium negative correlation between the mPHQ-15 and WHO-5, *r*(1977) = −0.27, *p* < 0.0001. Spearman’s correlation showed a small positive correlation between the GAD-7 and current feelings of insecurity, *r*(1977) = 0.246, *p* < 0.0001, and medium positive correlation with future fears, *r*(1979) = 0.292, *p* < 0.0001.

**Conclusion:**

The Gaza conflict impacted the psychological health of the Kuwait population, who shared geographical, geocultural, and geopolitical factors with those in the conflict zone. A global call to address mental health intervention programs for the public in and around conflict zones is a priority.

## Introduction

The ramifications of armed conflicts on the populations involved are well established. Conflicts between countries inevitably result in widespread human suffering, impacting families, health, the economy, and the environment. The effects of conflicts on health can include both physical and psychological harm to civilian populations within the conflict zone (1, 2). However, the impact of conflicts on health-related issues in populations outside the conflict zone requires further investigation.

In a review of mind–body interaction, Mallorquí-Bagué and colleagues (3) define anxiety as an emotional state influenced by psychological, physical, and behavioral factors. The DSM-5 (4) categorizes anxiety disorders into twelve disorders, including generalized anxiety disorder (GAD). GAD is characterized by excessive and persistent worry regarding daily events over time, which can interfere with daily activities, impair social and occupational performance, and disrupt relationships with others. The primary symptoms of GAD encompass restlessness or being on edge, easily becoming fatigued, difficulty concentrating, irritability, muscle tension, and sleep disturbances. These symptoms must be present independently of any medical conditions, mental health disorders, or substance use.

Research studies have found a positive relationship between GAD and the presence of somatic symptoms, as well as a negative relationship between GAD and wellbeing in various contexts, including hospitalization, natural disasters, COVID-19, and conflicts (5–13). Somatic symptoms refer to physical sensations that may disrupt daily functioning, such as headaches, musculoskeletal pain, abdominal aches, trouble sleeping, and fatigue, which cannot be explained by medical conditions (9, 14, 15). On the other hand, wellbeing is a holistic positive state encompassing emotions, free from anxiety and depression. The World Health Organization (WHO) considers wellbeing as a determinant of an individual’s and society’s health, influenced by multiple factors such as physical, environmental, social, emotional, psychological, activity participation, economic, and resilience (16–18).

Most studies on conflict primarily investigate health-related issues, such as mental disorders, somatic symptoms, and wellbeing, among populations within the conflict zones (19, 20). However, health-related issues among populations around, near, or within the geographical area of the conflict zones are rarely explored (12, 13, 21). There are various factors that may influence the health of these populations, including social media platforms, news, economic, environmental, cultural, and political ties to the conflict zones. Therefore, the objective of the study was to examine generalized anxiety disorder, associated somatic symptoms, and wellbeing among the Kuwait population during the Gaza Conflict of 2023, while also exploring the provoking factors.

Kuwait, with an area of 17,820 square kilometers (6,880 square miles), has a population of over 4.9 million, of which 1.5 million (32%) are Kuwaiti (22). Geographically located in West Asia and geopolitically in the Middle East, Kuwait adopts Arabic as its official language and Islam as its official religion. It lies in the northwestern part of the Arabian Gulf, bordered by Iraq to the northwest and Saudi Arabia to the south and southwest. The distance between Kuwait and the Gaza Conflict is approximately 1,270 kilometers (789 miles), crossing south of Iraq, north of Saudi Arabia, and Jordan. Kuwait shares geographical, geocultural, and geopolitical factors with the people in the conflict zone, including location, trade, political alliances, religion, language, and culture. The Gaza Conflict commenced on October 7th, 2023.

## Methods

### Study design

This study employed a descriptive correlational cross-sectional design. Variables related to anxiety, somatic symptoms, and wellbeing were self-reported and measured using ordinal scales.

### Participants

The target population comprised people living in Kuwait during the Gaza conflict. Snowball sampling was used to recruit the participants. Since the topic is rarely documented and the first of its kind in the region, snowball sampling is effective in reaching a wide range of the population. In addition, snowball sampling allows us to attain diverse perspectives and experiences. The inclusion criteria were age 18 years and older with all nationalities, races, educational levels, social status, and work standing. Exclusion criteria included being diagnosed with a mental health condition such as anxiety, depression, bipolar disorder, and mood disorders; chronic physical conditions such as chronic pain, arthritis, migraine (chronic headache), osteoporosis, herniated disk, and chronic irritable bowel syndrome; lung diseases such as asthma; or chronic fatigue. Participants were excluded if they reported any health conditions or disorders.

### Data collection tools

The generalized anxiety disorder assessment (GAD-7) is a self-reported screening tool. GAD-7 is designed to identify the severity of anxiety in adults 18 years and older, reflecting the DSM-5 criteria for generalized anxiety disorder. The tool includes seven items on a 4-point Likert scale (not at all = 0, several days = 1, more than half of the days = 2, and nearly every day = 3). The scores range from 0 to 21 (Minimal = 0–4, Mild = 5–9, Moderate = 10–14, and Severe = 15–21). A diagnosis of anxiety was considered at a score of 8 or higher (23, 24). GAD-7 has good internal consistency and convergent validity with Cronbach’s alpha above 0.82 and a cutoff point 8 for sensitivity and specificity (25). The Arabic version, validated and adapted by Alhadi et al. (26), was utilized for this study.

The Patient Health Questionnaire – 15 (PHQ-15) is a self-report screening instrument for somatic symptoms. The PHQ-15 comprises 15 items related to somatic symptoms, including stomach pain, headache, back pain, chest pain, and trouble sleeping. The items were scored on a 3-point Likert scale (not bothered at all = 0, bothered a little = 1, and bothered a lot = 2). Scores ranged from 0 to 30 (Minimal = 0–4, Mild = 5–9, Moderate = 10–14, and Severe = 15–30) (27). However, owing to cultural sensitivities, one item (question #11: pain or problems during sexual intercourse) was removed from the questionnaire due to cultural considerations and its limited relevance to the study population. While this modification may slightly alter the structure of the original scale, steps were taken to ensure the remaining items sufficiently captured the intended domains. However, it is acknowledged that this adjustment may affect the comparability of findings with studies using the unmodified version. Future research should consider revalidating the modified scale within this population. After adjustment, the modified PHQ-15 (mPHQ-15) comprises 14 somatic symptoms. The total mPHQ-15 score ranges from 0 to 28, with minimal (0–3), mild (4–8), moderate (9–13), and severe (14–28). Clinical and occupational healthcare settings have demonstrated high reliability and validity for the PHQ-15 (27). The Arabic version, validated and adapted by Alhadi et al. (26), was utilized for this study.

The WHO-5 is a widely recognized instrument for assessing subjective psychological wellbeing, with acceptable sensitivity (0.86) and specificity (0.81). It is a self-administered measure of wellbeing. The WHO-5 comprises five positively worded items scored on a 6-point Likert scale (at no time = 0 to all of the time = 5). The raw score was multiplied by 4 and transformed into a score from 0 to 100, with lower scores indicating worse wellbeing. A score of ≤50 indicates poor wellbeing and a score of 28 or below is indicative of depression (28, 29). However, the developers of the WHO-5 did not elaborate much on the scores between ≥51 to 100, besides that a 100 is the best possible mental wellbeing. Therefore, for better clarity and understanding, the authors in this study further described a score of 51–70 as fair, 71–85 as good, and 86–100 as excellent wellbeing. The Arabic version, validated and adapted by Ohaeri and Awadalla (30), was used in this study.

### Procedure

Ethical approval was obtained from the Institutional Review Boards of Kuwait University – Health Science Center (VDR/EC – 496. Nov. 19, 2023). The English and Arabic online survey, including the demographic questions, GAD-7, mPHQ-15, and WHO-5, was created using Google Forms. The link to the online survey included the title of the study, a brief description, and an agreement to participate. Clicking on the start icon indicates consent to participate. The link is sent to colleagues, family members, and friends via various social media platforms. The participants were asked to forward the link to others after completing the survey using a snowball sampling technique. Three days later, the survey was sent to new groups to reach more participants. Data collection began on November 20, 2023 and ended on November 27, 2023. This research protocol complied with the tenets of the Declaration of Helsinki.

### Data analysis

The Statistical Package for the Social Sciences (SPSS) version 28 was used for the analysis. Descriptive statistics were used to summarize the demographic and questionnaire results. Spearman’s Correlation and chi-square tests were used to determine the relationships between the variables. The choice of Spearman’s correlation as a non-parametric statistical test was specifically made due to its ability to measure monotonic relationships without requiring the assumption of normality in the data. Given the potential for non-linear relationships or non-normally distributed variables in this study, Spearman’s correlation was deemed more appropriate than Pearson’s correlation, which assumes linearity and normal distribution. Cramer’s V-test was used to assess the strength of the association. A value of *p* ≤ 0.05 was set as the threshold for significance.

## Results

Two thousand seventy-three (2,273) participants completed the survey. Two hundred and ninety-four surveys were excluded from the study because the participants were under the age of 18 and had mental disorders or chronic physical conditions. The final inclusion was 1979 participants. Of these, 1,582 (80%) were females, and 397 (20%) were males. The ages ranged from 18 to 89, with a mean of 34.89 ± 13.04 years (Table 1).

**Table 1:** Demographic description.

	Freq.	%
Nationality		
Kuwaiti	1,663	84
Non-Kuwaiti	316	16
Social status		
Single	802	41
Married	1,030	52
Divorced	123	6
Widow	24	1
Having children		
Yes	1,029	52
No	950	48
Work status		
Student	592	30
Employed	1,068	54
Unemployed	105	5
Retired	214	11
Education		
Some education	71	4
High school	326	16
Bachelor	1,371	69
Higher education	211	11
Exercise		
No	952	48
Yes	1,027	52
1–2 days	942	48
≥3 days	85	4
Family in conflict zone		
Yes	143	7
No	1836	93

**Figure 1:**
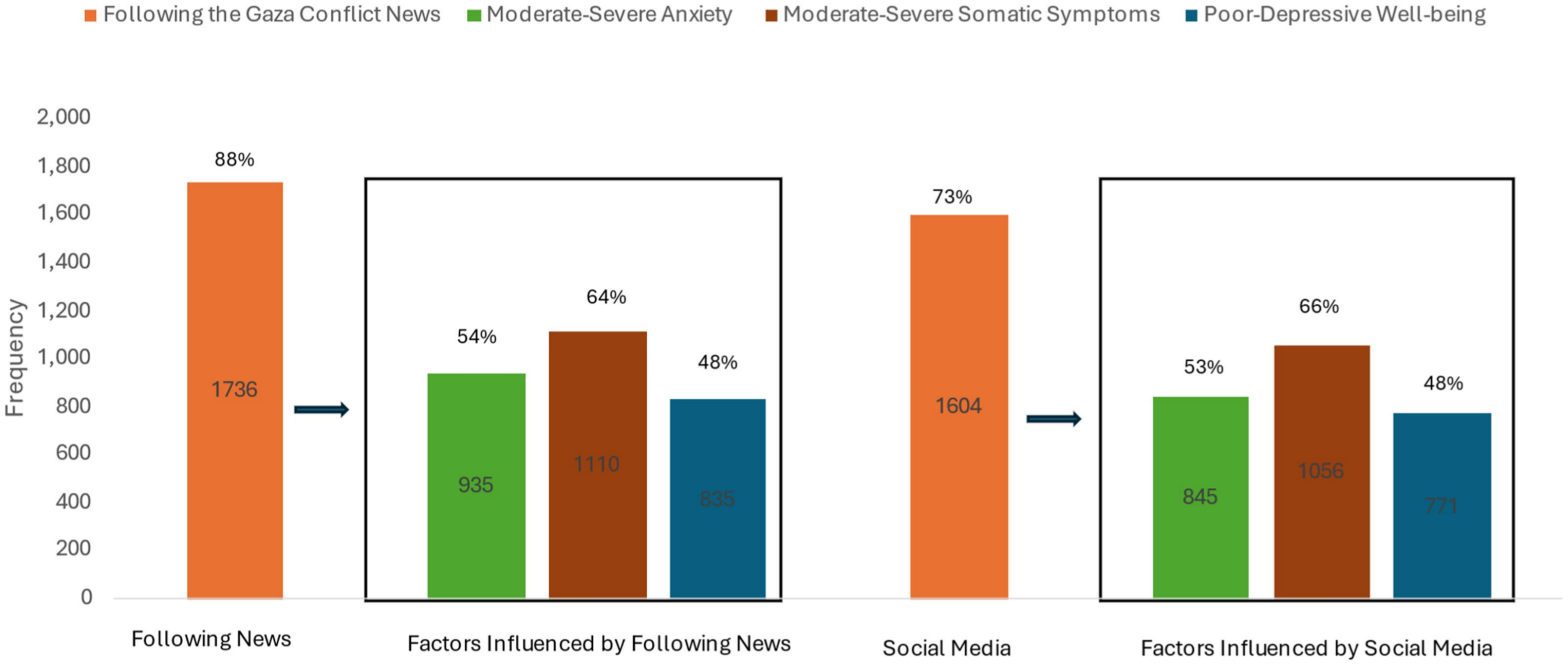
The influence of following the news and social media of the Gaza Conflict on anxiety, somatic symptoms, and wellbeing.

**Figure 2:**
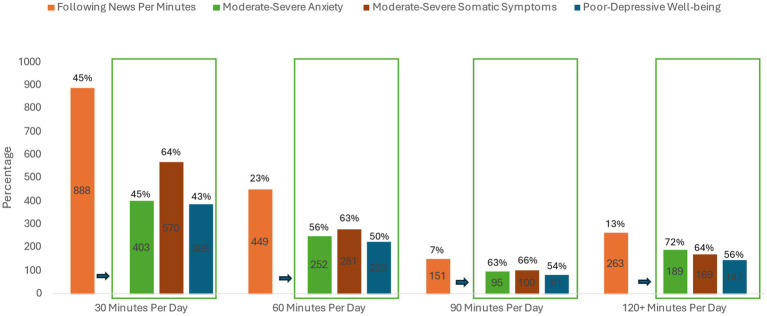
The influence of monitoring duration of the Gaza Conflict news on anxiety, somatic symptoms, and wellbeing.

**Figure 3:**
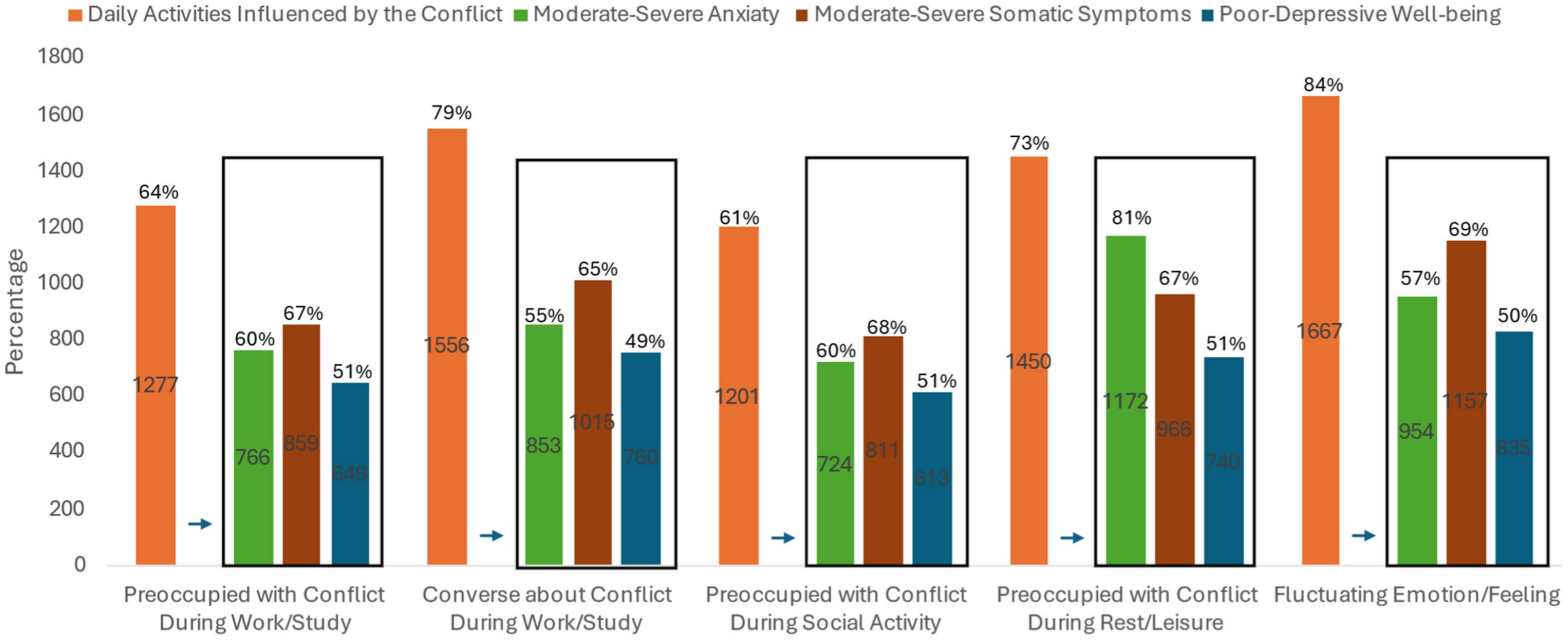
The influence of the Gaza Conflict on daily activities and emotions.

**Figure 4:**
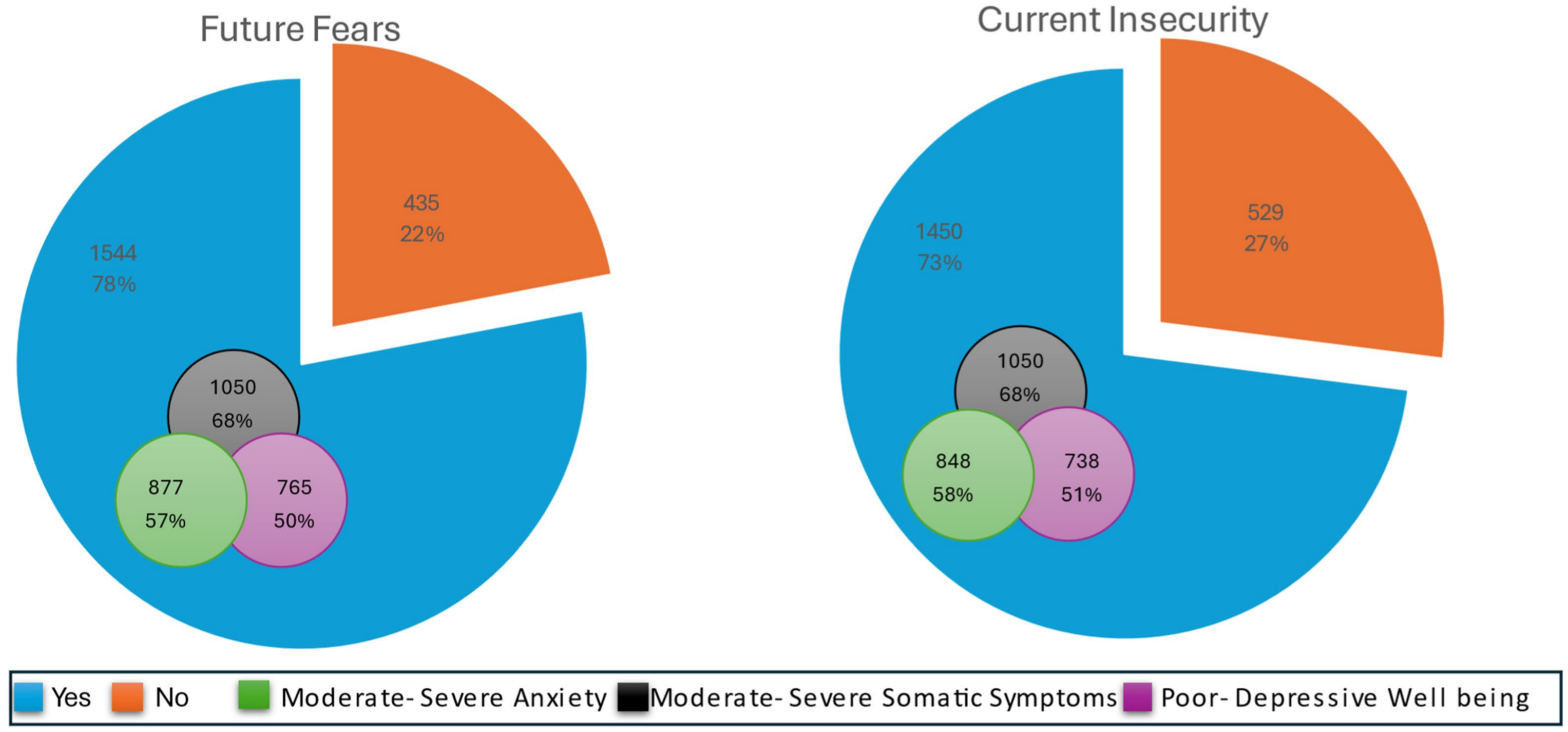
The influence of the Gaza Conflict on current insecurity and future fears.

Regarding the Gaza conflict, 88% of the participants indicated following news, in which 73% reported mainly following the news through social media platforms. Due to the association between anxiety, somatic symptoms, and wellbeing, most of those participants experience moderate to severe anxiety and somatic symptoms and poor to depressive wellbeing (Figure 1). Only, 12% of the participants were not interested in following the Gaza Conflict compared to 88% at least spent 30 min per day. Within all the duration categories of following the conflict news (30, 60, 90, 120 min), participants experience high percentages of moderate to severe anxieties and somatic symptoms and poor to depressive wellbeing (Figure 2). Moreover, more than 60% of the participants reported being preoccupied with the Gaza Conflict during work, social activities, and rest/leisure activities. In addition, 84% of participants described fluctuating emotions and feelings throughout the day. Over again, those participants experience high levels of anxieties, somatic symptoms, and poor to depressive wellbeing (Figure 3). Most of the participants (>70%) expressed a moderate level of current insecurity (*μ* = 5.29 ± 2.89) and future fears (*μ* = 5.35 ± 3.01) with high percentages of anxieties, somatic symptoms, and poor to depressive wellbeing (Figure 4).

The participants reported moderate anxiety and somatic symptoms on GAD-7 (*μ* = 10.20 ± 4.48 with CI (95%) = 10.00–10.40) and mPHQ-15 (*μ* = 11.12 ± 6.39 with CI (95%) = 10.84–11.40), and they exhibited fair wellbeing (*μ* = 53.35 ± 21.82 with CI (95%) = 52.39–54.31). Table 2 shows the GAD-7, mPHQ-15, and WHO-5 scores. Spearman’s correlation coefficient showed a large positive correlation between the GAD-7 and mPHQ-15 (*r*(1977) = 0.52, *p* < 0.0001). Therefore, the effect size for anxiety (*r^2^* = 0.27) indicated that a large portion of the variability in somatic symptoms (27%) was accounted for by the level of anxiety that the participants experienced. Also, Spearman’s correlation coefficient showed a medium negative correlation between the GAD-7 and WHO-5 (*r*(1977) = −0.36, *p* < 0.0001) with the medium effect size *r^2^* = 0.13 (13%), and a medium negative correlation between the mPHQ-15 and WHO-5 (*r*(1977) = −0.27, *p* < 0.0001) with the effect size *r^2^* = 0.07 (7%). Spearman’s correlation coefficient showed a small positive correlation between the GAD-7 and current feelings of insecurity, *r*(1977) = 0.25, *p* < 0.0001 with the effect size *r^2^* = 0.06 (6%), and a medium positive correlation between GAD-7 and future fears, *r*(1979) = 0.30, *p* < 0.0001 with the small effect size *r^2^* = 0.09 (9%).

**Table 2:** Participants’ descriptive statistics on GAD-7, PHQ-15, and WHO-5.

	Freq.	%	μ±	
**GAD-7**			10.20 ± 4.48	Moderate
Minimal (0–4)	220	11		
Mild (5–9)	740	37		
Moderate (10–14)	649	33		
Severe (15–21)	370	19		
**mPHQ-15**			11.12 ± 6.39	Moderate
Minimal (0–3)	312	16		
Mild (4–8)	409	20		
Moderate (9–13)	492	25		
Severe (14–28)	766	39		
**WHO-5**			53.35 ± 21.82	Fair
Depressed (≤28)	289	15		
Poor (29–50)	628	32		
Fair (51–70)	554	28		
Good (71–85)	401	20		
Excellent (86–100)	107	5		

The findings revealed a medium significant association between the variable fluctuation of emotions and feelings throughout the day and GAD-7 levels (*Χ^2^*(3) = 319.67, *p* < 0.0001, *V* = 0.402), a medium significant association with mPHQ-15 levels (*Χ^2^*(3) = 218.47, *p* < 0.0001, *V* = 0.332), and a small significant association with WHO-5 levels (*Χ^2^*(4) = 93.14, *p* < 0.0001, *V* = 0.217). Also, the findings revealed a medium significant association between the variable current feelings of insecurity due to the Gaza Conflict and GAD-7 levels (*Χ^2^*(3) = 193.06, *p* < 0.0001, *V* = 0.312), a small significant association with mPHQ-15 levels (*Χ^2^*(3) = 110.15, *p* < 0.0001, *V* = 0.236), and a small significant association with WHO-5 levels (*Χ^2^*(4) = 55.96, *p* < 0.0001, *V* = 0.168). Moreover, the findings revealed a medium significant association between the variable gender and mPHQ-15 levels (*Χ^2^*(3) = 254.67, *p* < 0.0001, *V* = 0.359) (Table 3).

**Table 3:** Association of the demographic factors with GAD-7, PHQ-15, and WHO-5.

Variables	GAD-7	mPHQ-15	WHO-5
Sex	*p* < 0.0001^**^, *V* = 0.167	***p* < 0.0001** ^**^ **, *V* = .359** ^**m**^	*p* < 0.0001^**^, *V* = 0.146
Nationality	*p* < 0.0001^**^, *V* = 0.095	*p* = 0.043^*^, *V* = 0.064	*p* < 0.0001^**^, *V* = 0.145
Social status	*p* = 0.164, *V* = 0.047	*p* < 0.0001^**^, *V* = 0.103	*p* < 0.0001^**^, *V* = 0.103
Having children	*p* = 0.031^*^, *V* = 0.067	*p* < 0.0001^**^, *V* = 0.154	*p* < 0.0001^**^, *V* = 0.159
Work status	*p* < 0.0001^**^, *V* = 0.077	*p* < 0.0001^**^, *V* = 0.133	*p* < 0.0001^**^, *V* = 0.110
Education status	*p* = 0.016^*^, *V* = 0.059	*p* < 0.0001^**^, *V* = 0.076	*p* = 0.479, *V* = 0.044
Exercise	*p* = 0.068, *V* = 0.054	p = 0.016^*^, *V* = 0.063	*p* < 0.0001^**^, *V* = 0.114
Following news	*p* < 0.0001^**^, *V* = 0.146	*p* < 0.0001^**^, *V* = 0.096	*p* < 0.0001^**^, *V* = 0.125
Duration of news	*p* < 0.0001^**^, *V* = 0.155	*p* = 0.004^*^, *V* = 0.070	*p* < 0.0001^**^, *V* = 0.095
Family in conflict zone	*p* < 0.0001^**^, *V* = 0.151	*p* = 0.072, *V* = 0.059	*p* = 0.002^*^, *V* = 0.092
Preoccupied during work	*p* < 0.0001^**^, *V* = 0.265	*p* < 0.0001^**^, *V* = 0.140	*p* < 0.0001^**^, *V* = 0.143
Converse during work	*p* < 0.0001^**^, *V* = 0.165	*p* < 0.0001^**^, *V* = 0.103	*p* < 0.0001^*^, *V* = 0.119
Converse during social	*p* < 0.0001^**^, *V* = 0.251	*p* < 0.0001^**^, *V* = 0.128	*p* < 0.0001^**^, *V* = 0.133
Preoccupied during rest	*p* < 0.0001^**^, *V* = 0.270	*p* < 0.0001^**^, *V* = 0.149	*p* < 0.0001^**^, *V* = 0.182
Fluctuating emotions	***p* < 0.0001** ^**^ **, *V* = .402** ^**m**^	***p* < 0.0001** ^**^ **, *V* = .332** ^**m**^	*p* < 0.0001^**^, *V* = 0.217
Current insecurity	***p* < 0.0001** ^**^ **, *V* = .312** ^**m**^	*p* < 0.0001^**^, *V* = 0.236	*p* < 0.0001^**^, *V* = 0.168
Future fears	*p* < 0.0001^**^, *V* = 0.279	*p* < 0.0001^**^, *V* = 0.205	*p* < 0.0001^**^, *V* = 0.151

^*^Significant at *p* ≤ 0.05; ^**^Significant at *p* ≤ 0.0001; m: Medium strength association between demographic factors and GAD-7, PHQ-15, and WHO-5.

## Discussion

Anxiety has been associated with war among people living in the conflict zone (20). However, anxiety has been rarely investigated among people living in the region around or near the conflict zone. This study aimed to explore anxiety and risk factors due to the Gaza conflict among people living in Kuwait.

This study highlights that participants experienced moderate levels of generalized anxiety disorder (GAD), with 52% reporting moderate to severe anxiety symptoms. The most common indicators, as measured by the GAD-7, included feeling nervous, anxious, or on edge; excessive worrying; difficulty relaxing; and becoming easily annoyed. These findings are consistent with prior research linking GAD to somatic symptoms, diminished wellbeing, feelings of insecurity, and future fears.

The interpretation of these results relies on the DSM-IV criteria, which were chosen due to their longstanding utility in identifying and categorizing anxiety severity in both clinical and research settings. Despite the availability of DSM-V criteria, DSM-IV remains a valid reference in contexts where established benchmarks are essential for comparing historical data or aligning with widely used assessment tools, such as the GAD-7.

From a clinical perspective, moderate-to-severe anxiety, as defined by the DSM-IV, can significantly impact social and occupational functioning. This underscores the importance of addressing the potential challenges faced by participants, including impaired interpersonal relationships and reduced productivity. Professional medical interventions, such as cognitive-behavioral therapy or pharmacological treatment, are recommended for those experiencing severe anxiety symptoms to improve quality of life and mitigate the risk of long-term complications. The association found in this study between the GAD-7 and the duration of following news about the Gaza Conflict, current feelings of insecurity, and future fears may explain participants’ anxiety, such as feeling anxious and afraid, worrying too much, annoyance, and irritation. Moreover, the National Institute of Mental Health (31) highlights that GAD interferes with activities of daily living, such as work/school performance, productivity, and relationships. Similarly, our findings showed that most participants were preoccupied with the Gaza Conflict during work and study, social activities, and rest and leisure. In addition, many participants (88%) followed the news on the Gaza Conflict, with 20% spending over 90 min a day following the news on the conflict. Likewise, Khansa and colleagues (32) emphasized that increased anxiety is associated with work impairment, diminished work productivity, and poor quality of life.

We believe that the smartphones’ easy and timeless use plays an influential role in continuous access to all sorts of written and visual news, information, and content about a conflict. Terry and colleagues (33) conclude that during public crises, activities and communications on social media platforms intensify to the point of affecting public health. Greenglass et al. (12), Malecki et al. (13), Riad et al. (21), and Mărcău et al. (34) underlined that Russia-Ukraine war news increased anxiety symptoms among adult populations in Germany, Poland, the Czech Republic, and Romania. In addition, Mărcău et al. (34) and Mejia and colleagues (35) highlighted that people were experiencing severe anxiety, depression, and stress, which were associated with the fear of breaking out of world war or using nuclear weapons during the Russia-Ukraine conflict. Additionally, the spread of fake news, misinformation, and propaganda on news and social media platforms can exacerbate feelings of fear, anger, worry, and frustration. Continuing the risk of daily following the news, preoccupation with the region’s insecurity, and fear of future expansion of the conflict might negatively affect work–study performance, productivity, and relationships. Levin et al. (20) discussed that continued and increased anxiety symptoms in a dose–response manner did limit the performance in daily activities. Examining the continuance of anxiety, Damsa et al. (36) and Pejic et al. (37), through neuroimaging studies, highlighted that the amygdala, anterior cingulate cortex, and insula may contribute to the progression and persistence of anxiety symptoms in individuals.

Based on the mPHQ-15 results, most participants experienced moderate-to-severe somatic symptoms. The most reported somatic symptoms were stomach pain, back pain, headaches, fatigue, low energy, trouble sleeping, and menstrual cramps. Moreover, most of the participants fell at the lower levels of wellbeing, with an average (=54) borderline to poor (≤50). Sleep troubles, restlessness, tension, and finding fewer exciting things to do were most frequently reported in the assessment of wellbeing. In the present study, an increase in GAD was associated with an increase in somatic symptoms and a decrease in wellbeing.

The DSM-V (4), NIMH (38), and National Health Services (NHS) (39) describe several physical symptoms to identify anxiety, including headaches, stomachaches, muscle tension, sleep disturbances, pain, fatigue, dizziness, and shortness of breath, which correspond to the somatic symptoms of the mPHQ-15. Using these two diagnostic frameworks together enhances the depth and applicability of the findings. The DSM-V criteria anchor the study in a structured diagnostic tradition, while the NIMH guidelines provide a lens for interpreting the complexities of anxiety in the participants. The relationship between anxiety and the somatic symptoms as well as wellbeing in our study paralleled the findings in other studies during natural disasters, COVID-19, and conflicts (5, 9, 11, 40–45). This relationship supports the biopsychosocial model of health, which posits that psychological factors can significantly impact physical health. Furthermore, this relationship is in line with the WHO’s definition of health, which includes not only the absence of disease but also a state of complete physical, mental, and social wellbeing (16). Moreover, high engagement with news about the conflict, as reported by 88% of participants, is a notable factor that might contribute to the psychological burden experienced by the participants. In addition, more than 60% of the participants were preoccupied with conflicts during work, social, and leisure activities, which could significantly disrupt their daily functioning and quality of life. The relationship found between factors, such as the duration of following the news, preoccupation with the conflict, fluctuation of emotions, current feelings of insecurity, and future fears with the PHQ-15 and WHO-5, could negatively influence the somatic symptoms and wellbeing of the participants. Even though the participants live far away, it is possible that shared Middle Eastern geocultural characteristics, such as language, religion, race, ethnicity, culture, and region, played a crucial role in relating and sympathizing with people in the conflict zone, which might have given rise to GAD and its association with the emergence of somatic symptoms and decline in wellbeing.

The Spearman correlations calculated between GAD-7, mPHQ-15, and WHO-5 provide valuable insights into the relationships among anxiety, somatic symptoms, and wellbeing. However, the discussion could benefit from a deeper exploration of why specific correlations were small or medium. For instance, the observed medium correlation between somatic symptoms and wellbeing might be contextualized within the broader literature, which suggests that somatic symptoms can manifest independently of subjective wellbeing levels due to cultural, psychosocial, or physiological factors (46). Such findings underscore the complexity of these relationships and highlight the need for more significant investigations that consider potential mediators or moderators.

The discussion on the causality between media consumption and increased anxiety should also be clarified. Given the cross-sectional nature of the study, causality cannot be inferred, as the temporal sequence of events is not established. While media consumption may be associated with heightened anxiety levels, the directionality and underlying mechanisms require further investigation through longitudinal or experimental designs. Furthermore, the role of social media in exacerbating anxiety warrants expanded discussion. Misinformation or sensationalized content on social platforms can amplify fear and uncertainty, particularly during crises, contributing to the heightened prevalence of anxiety symptoms. This phenomenon aligns with existing literature highlighting the detrimental psychological impact of media sensationalism. Addressing this issue through targeted public health interventions, such as media literacy campaigns and the promotion of reliable information sources, could mitigate these effects. Policy recommendations aimed at regulating the dissemination of misinformation and encouraging responsible media practices should also be considered to safeguard public mental health.

### Implications and future studies

Haug and colleagues (47) found a significant relationship between anxiety and functional somatic symptoms. They highlighted that patients with such symptoms tended to overuse the healthcare system and have a high degree of disability and sickness compensation. Therefore, the public should be aware of the possible health ramifications and receive psychological counseling as early as possible during such conflicts. In addition, the identified correlation among anxiety, somatic symptoms, and wellbeing suggests that healthcare providers should adopt a holistic approach when treating individuals with conflict-related health issues. In addition, state institutions should screen for anxiety among workers and students, offer counseling and positive support, and promote resilience and adaptation strategies. The need for resilience education among the public as a means of future adaptation to local or global adversity is highly invaluable, as it contributes as a protective factor to minimize trauma-related psychological distress, improve coping strategies with trauma, and promote positive functional performance (48–51). It is imperative for policymakers, governmental agencies, and educational institutions to support research studies relevant to crises encountered in a global context. Hence, a comprehensive and sustained global response is high priority for fostering public resilience and ensuring global peace, security, and mental health support (52).

Interestingly, Bohman and colleagues (53) concluded that somatic symptoms in young individuals may predict severe mental illness in the future. This study included many young participants. Therefore, early intervention is crucial for preventing mental health issues later in life. Additionally, the use of soft technology may be important in counseling. Ganesan and colleagues (54) highlighted a significant decrease in anxiety after engaging participants in tele-counseling during COVID-19. We also recommend that public health institutions generate health guidelines and procedures as a precaution against future conflicts to promote public health and wellbeing. Moreover, the pervasive nature of news consumption and its association with increased anxiety and somatic symptoms highlights the need for public health strategies to manage the psychological impact of media exposure during conflicts. This could include developing guidelines for media consumption and public education regarding coping strategies. The findings of the current study highlight the need for public health interventions and policy adaptations, including (1) The development of accessible counseling services and emergency psychiatric care are imperative, particularly in areas with high levels of conflict exposure; (2) Implementing community support programs can offer essential social support and contribute to the destigmatization of mental health issues. These programs facilitate community resilience and offer coping mechanisms for those affected; (3) Policymakers focus on allocating funds and resources to these programs in addition to pushing for mental health education and training; (4) Emergency response plans should integrate psychological support and physical health resources; and (5) It is critical to establish a national monitoring system to continuously assess the mental health status of the populations affected by conflicts. This system could help identify the emergence of mental health crises and the effectiveness of interventions over time. Thus, we recommend governmental initiatives that promote public awareness, periodical screening measures, and formal support for researchers in the field of public health promotion (55). We also call on global organizations, such as the United Nations (UN) and WHO, to develop global mental health intervention programs for populations in and around a conflict zone. Programs should be designed to address holistic needs, provide support, and raise awareness of the importance of mental health in populations around conflict zones. To the best of our knowledge, this study is the first to address the psychological impact of the Gaza Conflict on the Middle Eastern public. Therefore, similar future studies in this region are recommended to confirm these findings and possibly support further implications.

Future studies should examine the different periods and longitudinal effects of conflict on the population’s levels of anxiety, somatic symptoms, and wellbeing. Studies may also physically monitor heart rate, blood pressure, and blood sugar throughout the day or during and away from the news to determine the effect of following a conflict. Moreover, comparative analyses of populations from different conflict zones can elucidate the diverse factors that influence mental health during and after a conflict. Additionally, it is vital to investigate the effectiveness of various therapeutic interventions in conflict-affected populations. Research should focus on developing tailored intervention strategies that are culturally sensitive and context-specific. In addition, qualitative studies would provide an in-depth understanding of an individual’s conflict experiences.

### Limitations

The study had some limitations. Different examination periods or longitudinal designs are likely to demonstrate whether the outcome of this cross-sectional design ceases, fluctuates, or continues. Moreover, the sample used in this study exhibited a significant gender imbalance, with 80% of participants identifying as female. This disproportion raises valid concerns about the generalizability of the findings, particularly regarding the experiences and responses of male participants. While this imbalance was acknowledged as a limitation, it is important to investigate how this demographic skew may have influenced the results. For instance, previous research suggests that females are more likely to report higher levels of anxiety and somatic symptoms compared to males, which could mean that the study’s findings may overestimate the prevalence or intensity of these issues in the general population (12). Future studies should aim for a more balanced gender distribution to ensure that the results more accurately reflect the experiences of all demographic groups within the Kuwait population.

Additionally, the use of snowball sampling in this study, while practical given the sensitive nature of the research topic, may have introduced selection bias. This method often relies on participants recruiting others from their social networks, which can result in a sample that is not entirely representative of the broader population. For instance, individuals within the same social networks may share similar socio-demographic characteristics or experiences, potentially leading to an overrepresentation of certain viewpoints or experiences. To mitigate this limitation, future research could adopt a multi-stage random sampling approach or use stratified sampling techniques to ensure a more comprehensive representation. Moreover, integrating recruitment strategies that target underrepresented groups, such as males or individuals from diverse socio-economic backgrounds, could further enhance the generalizability of future studies.

Additionally, the generalizability of the findings to other regions, populations, and conflicts is limited. The limitations of self-reporting may be influenced by the fact that individuals interested in the topic may be more willing to complete surveys. In addition, the closed-ended questions in the survey did not allow the participants to provide more information about their experiences. Although more than half of the population of Kuwait is non-Kuwaitis, only 16% of our non-Kuwaitis participated.

The study is also limited by the absence of physical health measures, such as heart rate or blood pressure, which could provide valuable physiological insights to complement the psychological findings. Including such objective biomarkers in future research could enhance the biopsychosocial understanding of anxiety and its associated symptoms, offering a more holistic perspective. Moreover, these measures could help delineate the interplay between physical health and psychological wellbeing, thereby addressing gaps in the current study design. Future studies are encouraged to integrate these physiological markers alongside self-reported measures to better capture the multifaceted nature of anxiety and its impact on wellbeing.

## Conclusion

Most participants reported moderate-to-severe anxiety, somatic symptoms, and borderline wellbeing. Significant associations were found between anxiety, somatic symptoms, and wellbeing. The duration of news following, current feelings of insecurity, future fear, and fluctuating emotions were the primary risk factors. Future studies investigating the effectiveness of various therapeutic interventions in conflict-affected populations are warranted. Therefore, the global call for public intervention programs that are culturally sensitive and context-specific is a priority.

## Data Availability

The raw data supporting the conclusions of this article will be made available by the authors, without undue reservation.
